# Orthodontic Retention—Protocols and Materials—A Questionnaire Pilot Study among Polish Practitioners

**DOI:** 10.3390/ma15020666

**Published:** 2022-01-16

**Authors:** Maciej Jedliński, Marta Mazur, Krzysztof Schmeidl, Katarzyna Grocholewicz, Roman Ardan, Joanna Janiszewska-Olszowska

**Affiliations:** 1 Department of Interdisciplinary Dentistry, Pomeranian Medical University in Szczecin, 70-111 Szczecin, Poland; krzysztof.schmeidl@gmail.com (K.S.); katgro@pum.edu.pl (K.G.); jjo@pum.edu.pl (J.J.-O.); 2 Department of Oral and Maxillofacial Sciences, Sapienza University of Rome, 00161 Rome, Italy; marta.mazur@uniroma1.it; 3 Department of Economic Sciences, Koszalin University of Technology, 75-343 Koszalin, Poland; roman.ardan@tu.koszalin.pl

**Keywords:** fixed retention, orthodontic materials, clinicians’ preferences, retention wire, Polish orthodontist, retention protocol, retainer, orthodontic retainer, stainless-steel wire, gold chain

## Abstract

The aim of the study was to analyze retention protocols and materials for fixed retainers used by clinicians providing orthodontic treatment in Poland. The survey was carried out from February to April 2021. The questionnaire was designed using the Google Forms tool. After validation, the questionnaire was delivered to verified active orthodontists gathered in a closed social media group of 615 members. Finally, 104 answers were received. Answers to individual questions were provided in percentages and tabularized. A chi-squared test of proportion was used to compare: the proportion of clinicians using retainers of different characteristics and the proportions of clinicians indicating the superiority of a given clinical solution. Rectangular steel braided wire was rated as most reliable. However, doctors who declared to use gold chain were mostly solely using this type of wire. Multistranded round wire was rated the worst. Fiber-reinforced composite was mainly used in periodontal patients. The protocols used by Polish orthodontic practitioners relied on double long-term retention with regular follow-up. The most popular material was stainless steel braided rectangular wire bonded with a flowable composite. Most clinicians believed they could maintain the treatment results, but they declared that patients’ cooperation was a challenge.

## 1. Introduction

Effective maintenance of tooth position achieved in the active phase of treatment is a prerequisite to consider orthodontic treatment successful [1]. However, perfect retention of new occlusal conditions is difficult to secure, more challenging than obtaining optimal results with an active appliance [2]. It is shown that the tendency to relapse is an individual trait [3]. Individual patients’ characteristics that may cause a malocclusion to relapse include: (i) long-lasting remodeling periodontal tissues; (ii) excessive force of the facial muscles; or (iii) changes caused by patients’ growth and aging [4]. Tooth movement resulting from aging occurs in all subjects, regardless of the history of orthodontic treatment [4]. An important factor is also the patient’s compliance, which usually decreases over time, regardless of the type of retention used [5]. Already, in 1969, Horowitz and Hixon pointed out the prevalence of the problem of malocclusion relapse [6], and this issue is still valid today [7]. Despite continuous scientific advances in orthodontics, the results of many questionnaire studies show that the retention phase remains a source of controversy among clinicians, and there is no consensus on which therapeutic measures should be taken and what protocol the orthodontist should follow, when the active phase of treatment is over [8,9,10,11]. There is also no common consensus on type of wire, bonding materials, and procedures. The current scientific evidence on this topic is insufficient [12]; thus, the choice of treatment protocol remains subjective [13]. Orthodontists have no influence on the patients’ biological characteristics and have a limited impact on their compliance; therefore, the protocol of fixed retention bonding should be adjusted based on that solutions that most ensure clinical success. Bonding a fixed retainer makes the true goal of the retention phase—maintenance of treatment results—less dependent on patient’s compliance [14], as well. The choice of material may influence the failure rate of the applied fixed retention [15]. Numerous studies can be found concerning the preferences of orthodontists referring to the retention protocol used or appliances used for removable retention [8,9,10,11,16,17,18,19]. In the current literature, single studies could be found regarding clinicians’ preferences in reference to materials used for fixed retention [20,21]. However, none of them focused on opinions about characteristics of each material, which led orthodontists to their clinical choices, but rather focused on characteristics of the patient. This seems confusing, considering the wide range of materials available on the market—wires, fiber-reinforced composite (FRC), and materials used to bond them to the tooth surface. Therefore, it seems reasonable to learn these opinions and understand the clinicians’ choices in reference to procedures and materials. Furthermore, it seems necessary to examine the preferences and opinions of orthodontists regarding the effectiveness of materials to bond fixed retention appliances. The objective of the study was to find out:

1. what protocols are introduced in the retention phase of orthodontic treatment; and
2. what materials are used to fabricate and bond fixed retainers by clinicians providing orthodontic treatment in Poland. The null hypothesis was that the orthodontic treatment providers use various materials basing on the individual experience.

## 2. Materials and Methods

The target population of this questionnaire was a group of specialists in orthodontics performing orthodontic treatment in Poland. Based on the latest systematic review—since no method nor choice of fixed retention has been scientifically proven to be superior to other materials, the questionnaire has been set to learn about clinical preferences of the orthodontic treatment providers [15], trying to find solutions based on the “wisdom of crowds”, in this case, orthodontists offering commercial treatment. The survey was carried out in Polish language (Supplementary S1) within 3 months, from February to April 2021. First, the questionnaire designed was verified by a group of ten experienced academic orthodontists from northern Poland, who were not participating in the final study group. Then, the questionnaire was delivered to active clinicians by publishing the link on groups in social media restricted to orthodontic specialists or post-graduate students in orthodontics (admission to a group involves authorization in the form of a medical practitioner public license number, thanks to which it is possible to check whether a given person is actually an orthodontist). The group “Lekarze Ortodonci” had 615 members. The questionnaire, consisting of 17 questions, was designed using the Google Forms (Google, Mountain View, CA, USA) tool. The survey was performed while maintaining the anonymity of the respondents. However, the Forms Tool was adjusted to require prior Google account verification in order to prevent multiple completion of the survey or sending of responses. The post was removed from the group 2 months later.

The questions were presented in Polish, and their translations are as follows:

1. What age group do you belong to?
  (a) <30
  (b) 30–40
  (c) 40–50
  (d) >50
2. What type of retention do you use in patients after the active phase of orthodontic treatment?
  (a) Only fixed retention
  (b) only removable retention
  (c) both fixed and removable retention
3. In your opinion, the retention phase after removing the fixed braces should last: (More than one answer allowed.)
  (a) Half of the period of active treatment
  (b) The same as the active treatment
  (c) 2 times longer than the active treatment
  (d) year
  (e) 2 years
  (f) 5 years
  (g) Lifelong
  (h) Another (please share your opinion):
4. The frequency of control visits with a retention appliance in my practice is:
  (a) Every month
  (b) Every 3 months
  (c) Every six months
  (d) Once a year
  (e) The first and second visit every 3 months, and then every six months
  (f) The first visit after a month, the second after 3 months, and then every six months
  (g) Another (please share your opinion):
5. What method do you use to bond retainers? (More than one answer allowed.)
  (a) I bond them directly
  (b) I bond them indirectly
6. How do you evaluate the results of the retention treatment used? (More than one answer allowed—without contradictory answers.)
  (a) Maintaining the perfect position of the teeth is difficult.
  (b) I am able to perfectly maintain the results of the active phase of treatment in most patients
  (c) Fixed retention failures are a serious clinical problem.
  (d) Fixed retention failures are a marginal clinical problem.
  (e) Patients usually cooperate during the retention phase of orthodontic treatment.
  (f) Patients usually fail to cooperate during the retention phase of orthodontic treatment.
7. What kind of fixed retention do you use? (More than one answer allowed.)
  (a) Fiber Reinforced Composite
  (b) Steel wire
  (c) Titanium wire
  (d) I do not use fixed retention
  (e) Another (please share your opinion):
8. If you use the fiber-reinforced composite, in what form do you use it?
  (a) tape
  (b) knot
9. If you use wire, what kind of material is it? (More than one answer allowed.)
  (a) Single steel wire
  (b) Multistranded round steel
  (c) Rectangular steel braided wire
  (d) Titanium wire
  (e) Golden chain
  (f) Nickel titanium wire
  (g) Another (please share your opinion):
10. If you use a wire, what are its dimensions?
  (a) 0.014″ × 0.014″
  (b) 0.015″
  (c) 0.016″
  (d) 0.016″ × 0.022″
  (e) 0.0175″
  (f) 0.0195″
  (g) 0.027″
  (h) I do not know
  (i) Another (please share your opinion):
11. What kind of material do you use to bond retainer wires? (More than one answer allowed.)
  (a) A liquid composite material dedicated to retention appliances
  (b) A flowable composite material for restorations
  (c) Composite condensable material intended for restorations
  (d) Light-curing adhesive for orthodontic brackets
  (e) Light-curing material intended for indirect bonding
  (f) A chemically hardened material intended for indirect bonding
  (g) Another (please share your opinion):
12. In your opinion, the multistranded round steel wire (More than one answer allowed—without contradictory answers.)
  (a) It is not always effective in preventing unwanted tooth displacement
  (b) Effectively prevents unwanted tooth displacement
  (c) It is easy to bend
  (d) It is hard to bend
  (e) It debonds often
  (f) It rarely debonds from the teeth
  (g) It is easy to bond
  (h) It deforms rarely
  (i) It deforms often
  (j) I have no opinion; I do not use it
13. In your opinion, the rectangular steel wire (More than one answer allowed—without contradictory answers.)
  (a) It is not always effective in preventing unwanted tooth displacement
  (b) Effectively prevents unwanted tooth displacement
  (c) It is easy to bend
  (d) It is hard to bend
  (e) It debonds often
  (f) It rarely debonds from the teeth
  (g) It is easy to bond
  (h) It deforms rarely
  (i) It deforms often
  (j) I have no opinion; I do not use it
14. In your opinion, the gold chain (More than one answer allowed—without contradictory answers.)
  (a) It is not always effective in preventing unwanted tooth displacement
  (b) Effectively prevents unwanted tooth displacement
  (c) It is easy to bend
  (d) It is hard to bend
  (e) It debonds often
  (f) It rarely debonds from the teeth
  (g) It is easy to bond
  (h) It deforms rarely
  (i) It deforms often
  (j) I have no opinion; I do not use it
15. When do you use Fiber Reinforced Composite?
  (a) I do not use it
  (b) I use it in patients with periodontal disease
  (c) I use it in most patients after orthodontic treatment
  (d) I use it in all patients
  (e) Another (please share your opinion):
16. What kind of material do you use to bond FRC splints? (More than one answer allowed—without contradictory answers.)
  (a) A liquid composite material dedicated to retention appliances
  (b) A flowable composite material for restorations
  (c) Composite condensable material intended for restorations
  (d) Light-curing adhesive for orthodontic brackets
  (e) Light-curing material intended for indirect bonding
  (f) A chemically hardened material intended for indirect bonding
  (g) Another (please share your opinion):
17. In your opinion—Fiber Reinforced Composite: (More than one answer allowed—without contradictory answers.)
  (h) It is aesthetic
  (i) It is durable
  (j) It is easy to bond
  (k) It deforms rarely
  (l) Effectively prevents unwanted tooth displacement
  (m) It hinders hygiene
  (n) It detaches easily
  (o) It’s hard to bond
  (p) It deforms often
  (q) It is not always effective in preventing unwanted tooth displacement
  (r) I have no opinion; I do not use it

Answers to individual questions were given in percentages and tabularized. Chi-squared test of proportion was used for three types of comparisons:

- Difference in proportion of clinicians compatible with the characteristics of the appliance.
- Difference in proportion of clinicians as to the superiority of a given clinical solution over others.

The difference in proportion of the number of positive and negative traits of the three types of wire was counted in order to determine the characteristic of different types of wires in the opinion of respondents. Difference was considered significant at *p* < 0.05. R statistical software (The R Foundation for Statistical Computing, Wirtschaftsuniversität Wien, Vienna, Austria) was used for the calculations [22].

## 3. Results

The raw results of the survey are presented in Supplement S2. The overall response rate was 16.9%, with a total of 104 responses for the 615 members of the group. According to Supreme Medical Council in Poland, there are 1296 clinically-active specialists in orthodontics [23], so the respondents of the study constitute a significant part of this group.

### 3.1. Personal Characteristics of the Respondents

**Figure 1:**
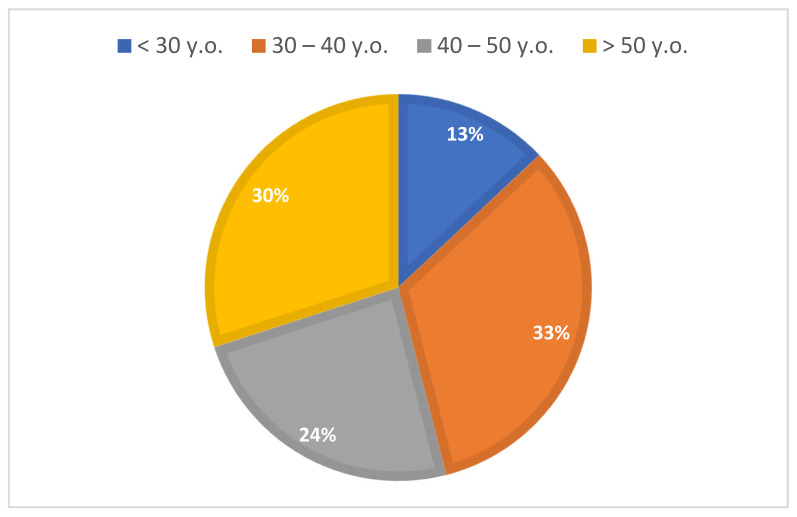
Distribution of the study group according to age.

The age structure of study group is presented in Figure 1.

### 3.2. Retention Protocol

**Figure 2:**
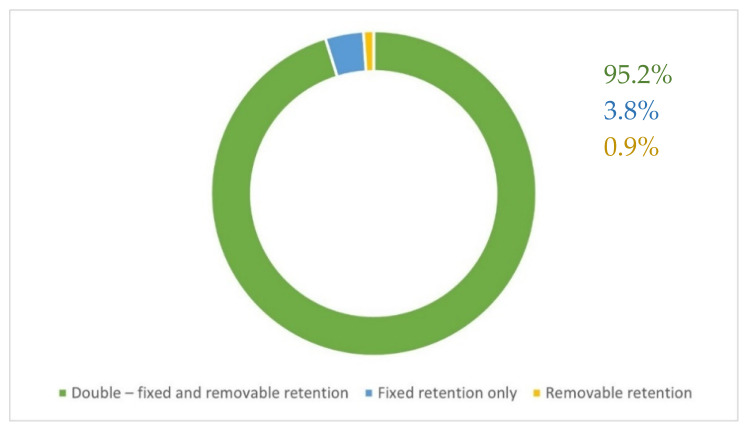
Distribution of the retention types used.

The type of retention used is presented in Figure 2. Most of the practitioners used double retention in all cases, and bonded fixed retention directly, rarely using indirect bonding technique.

**Figure 3:**
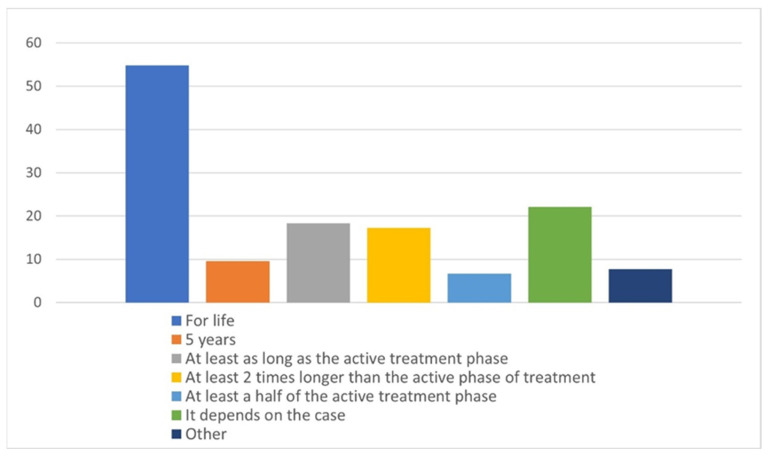
The duration of follow-up declared by the respondents.

Fifty-four percent of the respondents believe that the retention phase of orthodontic treatment should be lifelong, and 20% of the respondents provided answers different than those that could be chosen. Thus, the category “other” contains the following answers: 3 years—2 persons, 3–4 years—1 person, and at least 3 years—2 persons, whereas the other responses in this category differentiate retention duration between children and adults in different ways (Figure 3).

The follow-up protocol was also not consistent in the study group. However, neither option was clearly more popular. The follow-up appointments timetable among 44.2% clinicians was as follows: first visit after a month, the second after 3 months, and then every six months. Another protocol: the first and second visits every 3 months, then every six months, was followed by 29.8% practitioners, whereas 17.3% see their patients every 6 months and 8.7%—once a year.

Direct bonding was declared by 100% of the participants, but 7.7% also used indirect bonding.

Fifty-one and nine tenths percent of the respondents believed that they were able to perfectly maintain the results of the active phase of treatment in most patients. However, 48.9% of them considered maintaining the perfect position of the teeth to be challenging. Thirty-four percent stated that failure of fixed retention was a serious clinical problem, and 30.8% considered it as a marginal clinical problem. The doctors complained about poor cooperation of their patients. Fifty-five and two tenths percent responded that their patients usually fail to cooperate during the retention phase of orthodontic treatment, and 30.9% reported that it was difficult to objectively assess the effectiveness of retention appliances, as a large percentage of patients did not come for the control. Only 41.7% said that patients usually cooperated during the retention phase of orthodontic treatment.

### 3.3. Materials Used for Fixed Orthodontic Retention Appliances

The most popular type of fixed retention among Polish clinicians was stainless steel wire (used by 75.7%), followed by gold chain (GC)at 26.9%. Twenty-two and three tenths percent used fiber-reinforced composite (FRC), 19.2% used titanium wire, and 1% did not use any type of fixed orthodontic retention. When asking about the type of steel wire, 57.7% used braided rectangular wire (BRW), 26.9% used multistranded round wire (MRW), and 4.8% used single round steel wire. Within the group that uses FRC for retention, 90% used fiber in the form of tape, and 10% in the form of a knot.

Concerning the dimensions of wires used, 35.9% respondents admitted that they did not know the dimensions of the wire used, 27.2% answered 0.016″ × 0.022″, 0.0175”, and 0.027”, at 13% each. Thirty-two and six tenths percent wrote one of 11 different answers. However, none of them exceeded 3%.

When it comes to materials used to bond the wire, 63.7% declared that they used liquid composite material dedicated to retention appliances. However, the same percentage answered that they also used liquid composite material dedicated to dental restorations. Seven and seven tenths percent used condensable material manufactured for dental restorations, and 5.7% used chemical-cured material intended for indirect bonding.

When it comes to FRC retention, a vast majority (70.2%) of the respondents admitted that they did have any opinion, since they did not use it in any patients. Twenty-eight and eight tenths percent used it in periodontal patients, and 1% used it when a patient was allergic to metals. One respondent used it in all patients. Eighteen and four tenths percent agreed that FRC retention was aesthetic, 14.6% that it was durable, and 10.7% that it was easy to bond, the same percentage in which it effectively prevented unwanted tooth displacement, with 8.7% reporting it rarely deformed. However, 25.2% had the opinion that it compromised oral hygiene, 16.5% reported that it was difficult to bond, and 10.7% that it often debonded. Only 3.9% agreed to the statement that it often deformed.

In addition to the doctors’ preferences, it would be worth determining why they use a particular type of retention wire. Thus, they were asked to choose the statements which, in their opinion, best described the given wire. The null hypothesis of the study was confirmed. The percentage of answers to the questions is presented in Table 1.

The answer “rectangular steel wire is easy to bend” had significantly higher proportions than the two other types of wire. “Gold chain wire is easy to bend” and “gold chain is hard to bend” had significantly lower proportions than the two other types of wire (Table 2). It means that, overall, the orthodontists considered rectangular wire to be the easiest to bend, followed by the gold chain. They heavily criticized the round wire. However, the users of gold chain were of the opinion that it was the easiest to bend of all three types of wire, considering the other 3 types as hard to bend.

The rectangular wire has significantly higher proportions than two other types, while the round wire has significantly lower proportions than two other types of wire. However, it should be marked, that there is also a small group of orthodontists who believe MRW is harder to bend than GC (Table 3).

The rectangular wire had significantly higher proportions of answers than two other types, while the round wire has significantly lower proportions than two other types of wire (Table 4).

The gold chain had significantly higher proportions than two other types, while rectangular wire had significantly higher proportions than round one. The rectangular wire had significantly lower proportions in the answer “It detaches rarely from the teeth”, than two other types of wire, which means that it detaches more often (Table 5).

According to the respondents, gold chain deforms least frequently during follow-up. Round wire deforms more frequently than the other types of wire (Table 6).

## 4. Discussion

The presented study shows what protocols are used by Polish orthodontists in the retention phase, what features, according to Polish orthodontists, present various orthodontic wires, and how orthodontists fabricate fixed orthodontic retainers. It should be noted that, when it comes to materials for bonding fixed retention, Polish orthodontists trust their skills and follow the principle “it works in my hands”. This seems important from the point of view of the orthodontic materials sales market.

### 4.1. Study Group

The first question was used to determine the age group. Questions 2–6 were asked to provide information on the type of retention appliances used, the scope of the duration of the retention phase, and the frequency of follow-up visits. The latter questions referred to the type of fixed retention used, detailing the wire or FRC used, and the bonding material used. In questions 1, 2, and 4, selecting only one answer was possible, while, in questions 3 and 5–17, choosing multiple answers was allowed.

Sample size assessment was not possible in the present study because the authors were dealing with a multi-path analysis of non-parametric data, which includes the opinions of Polish clinicians concerning the use various materials for fixed retention appliances. According to the Supreme Medical Council in Poland, there are 1296 clinically active specialists in orthodontics [23]. Thus, the present study on 104 clinicians can be considered representative for all Polish orthodontic treatment providers, as they constitute more than 8% of the whole group.

### 4.2. Retention Protocols

The finding that almost all respondents used double retention, fixed and removable, is inconsistent with previous reports, where doctors who used double retention were in the minority of those surveyed. Most clinicians preferred to use removable retention [8,9,10,11,14,18,19]. Scientific evidence shows that it is permanent retention that ensures the best maintenance of treatment results for a longer period of time [24]. Interestingly, only recently, in light of new scientific evidence, have orthodontists begun to declare a routine use of fixed retention [8,14].

The use of double retention by most Polish practitioners confirms the high attention they pay to maintaining the results of the active treatment. Double retention is also gaining popularity outside Europe; however, it is still not a standard [25]. Retention type is usually based on the doctor’s clinical judgment and experience [9,10,11,19]. Factors influencing the final decision on the type of retention used include oral hygiene, case “severity”, type of malocclusion, and use of extraction during treatment, especially in maxilla [9,12,16,19]. However, Malaysian orthodontists do not believe that the use of extractions should have an impact on the type of retention used [10]. Interestingly, Al-Moghrabi et al. [26] pointed out that double retention was widely prescribed, even if little was known about its additional benefits, and it required more attention to oral hygiene. However, there are publications reporting that double retention for a prolonged period noticeably reduces tooth displacement, especially of the lower anterior teeth [27]. It is evident that there is a lack of high quality clinical scientific studies, especially randomized clinical trials or cohort studies, which makes it difficult to provide clinical recommendations based on scientific evidence.

Dental aesthetics is one of the key needs leading a patient to the dental chair, and high aesthetic demands are common among patients [28,29]. The number of lawsuits against orthodontists has dramatically increased in the recent years [30]. Therefore, it seems rational and economically justified to introduce double retention in everyday practice to maintain positions of the teeth achieved during the active phase of treatment. A fixed retainer and a thermally-formed splint are often recommended, since a fixed retainer holds the teeth in the anterior region while the patient is not wearing a removable retainer, and the splint is able to additionally protect the patient from displacement of posterior teeth (as well as anterior teeth in the event of fixed retention failure). In association with the results of the survey, it should be noted that, according to the respondents, the GC is the type of wire that detaches the least frequently. This can be very important for everyday orthodontic practice. A low probability of detachment allows the doctor to convince the patient of the effectiveness of this type of fixed retention, regardless of the physical characteristics of the wire, causing a potential risk to the stability of treatment outcomes in the future, known only to the doctor from scientific reports.

As far as the duration of retention is concerned, more than 50% of the practitioners declared recommending life-long retention to their patients. This approach is similar to that reported in the studies by Pratt et al. [16] and Padmos et al. [31]. On the other side, quite a high percentage of clinicians chose different retention periods: “at least as active treatment”, “at least two times longer as active treatment”, or “at least a half of the active treatment”. According to scientific evidence on the stability of orthodontic alignment, ceasing retention at any time does not guarantee stable orthodontic alignment [32]. Any malalignment of the anterior teeth may be esthetically unacceptable for the patient. On the other side, retention is associated with cost, discomfort, and potential iatrogenic effects [33], and it requires cooperation [34]. Thus, individual approach, as in “it depends on the case”, is also reflected in the present study.

In a 1984 Polish textbook on orthodontics by Łabiszewska-Jaruzelska, it is stated that a fixed appliance should be left passive in the mouth for several months, and then it should be replaced by a removable appliance; however, how long the retention phase should last is not recommended [35]. This statement seemed to influence the responses within the questionnaire.

The phrases: “at least as long as active treatment” and “at least a half of the active treatment”, describing the length of the retention period, come from another Polish orthodontic textbook, “Zarys współczesnej ortodoncji”, by Karłowska, published in 2016 [36], which is currently used in under- and postgraduate teaching. The mentioned recommendations on retention duration do not refer to fixed appliance treatment; they refer to prolonged use of functional appliances after achieving the desired effect of treatment. None of the two handbooks provides a source citation as scientific background for the recommendation of orthodontic retention. In the latest handbook edition [36], it is indicated that the retention phase after treatment with fixed appliances requires a longer period. Thus, it can be supposed that, due to a misunderstanding, some practitioners choose retention protocols not recommended to fixed but, rather, to removable appliance treatment.

Most respondents declared seeing their patients one month following debonding fixed appliances, then after 3 months, and then every 6 months. From the point of view of the scientific evidence, this approach seems optimal. The most frequently chosen answers indicated that clinicians know about the need of frequent controls during the first six-months period, which is scientifically valid [15]. A recent meta-analysis [15] showed that most failures of fixed retention occur within the first 6 months after bonding retainer, and, during that time the patient should be under frequent supervision. However, almost 30% of the clinicians declared recommending the first appointment with fixed retention after 3 months, and 17.3% after 6 months. This approach relies on the patient’s cooperation and “emergency” visits in case of a failure. Thus, the patient could be seen earlier and more often to avoid possible relapse of malocclusion. A similar approach to follow-up was described in the study by Padmos from 2018 [31]. However, in most of the survey papers in the literature on orthodontic retention [9,10,12,14,16,17,18,19], the authors pointed out the need for more frequent follow-up visits (at least four) during the first year. Further monitoring of retention tends to be delegated to general practitioners, or even left to the patient’s self-monitoring. Nevertheless, unwanted tooth movement has been shown to occur, despite proper adhesion of retainers in the oral cavity, especially in the maxilla [37]. This means that, if the patient was monitored long-term by the orthodontist, the latter, having the appropriate documentation, could detect these displacements earlier; thus, the orthodontist could introduce less aggressive and cheaper retreatment, for example, in the form of several aligners [38].

All the participants declared that they bonded fixed retainers directly. Since two answers were possible, indirect bonding was indicated by a small percentage of the respondents, as well. According to a recent systematic review [15], there is no difference concerning the frequency of failures referring to direct versus indirect bonding. However, indirect bonding saves chair time [39]. The reason why Polish orthodontists bond their retainers directly may be the desire to avoid the laboratory costs (which may not seem cost-effective). An interesting, recently reported novelty is finding application in fixed post-orthodontic retention bonding using 3D printed materials [40]. These devices, appropriately designed on a 3D virtual model, could greatly facilitate direct bonding of retention wires in the future.

### 4.3. Materials Used for Fixed Retention

Two survey studies could be found asking about the type of wire used; both the studies were performed by the same group of authors [8,31]. However, they asked only about type of wire chosen, not reasons for such a choice. The popularity of the material reported in this article was similar to that in the present study. Steel rectangular wire, steel round wire, and gold chain were the most popular, similar to that among Polish clinicians, wherein steel rectangular braided wire was also the most popular, followed by a gold chain and a round steel wire (the difference between the last two is statistically insignificant).

The fact that more than 50% of the practitioners believed that they could perfectly maintain the results of the active treatment may indicate that the clinicians believe in the effectiveness of retention appliances. On the other side, almost 50% agreed that maintaining perfect positions of the teeth was difficult. It is possible that the clinicians see the main reasons for their problems with retention in patients’ cooperation, since more than 50% declared that their patients fail to cooperate during the retention phase.

The diversity of wires used for fixed retention reflects a lack of clinical scientific evidence proving a lower failure rate of any wire [18,32]. Most of the survey studies drew attention to the lack of clear clinical guidelines and sought the guidelines on which type of retention should be applied [9,11,14].

Most clinicians using fiber-reinforced composite preferred a tape to a knot. No studies have been found in the literature that could be used for the comparison with this finding of the present questionnaire study. No studies have reported on a lower failure rate of a tape versus knot. However, tape may require less occlusal space and, therefore, is chosen more often. A recent report has shown that FRC retention can be as stable as the one obtained with steel wires [41]. FRC retention is more sensitive to operator skills, and, with imperfect bonding, the failure rate could be much higher [15]. As an example, when composite resin is fully polymerized, high-quality fiber strips strongly fuse with one another and, thus, can significantly increase the resin strength and durability by up to 300% compared to inaccurately polymerized material [42].

The most popular type of wire used in the present questionnaire is stainless steel braided wire, but nearly 36% of clinicians do not know its dimensions. It can be supposed that they buy marketed, popular retention wires available on the market and do not check their sizes nor the clinical characteristics of the material used. The respondents mentioned that stainless steel braided wire was easy to bend and bond, rarely detached from the teeth, and usually correctly maintained the position of the teeth. This is consistent with the findings of Arnold et al. [43], who reported that braided 0.016 × 0.022-inch stainless steel wire was torque resistant and should replace multistranded round wires. The use of multistranded round wires was declared by only 13% of the practitioners. The interviewed group believed that a round wire retainer was difficult to bend and bond, detached more often from the teeth, did not maintain the position of the teeth satisfactorily, and easily became deformed. It may indicate that most clinicians were familiar with reports on adverse effects described, including wire untwisting and torquing teeth [44,45]. Interestingly, doctors who use gold chains were the group that was most convinced to the material of their choice, and they most negatively assessed the other wire types. The comparison of the proportions in the responses showed that, according to the respondents, it is the gold chain that best maintains the position of the teeth. This is contrary to many in-vitro studies that indicate that gold chain wire has the lowest bending and torsional stiffness, and it is not resistant to the masticatory forces, which may lead to activation in long-term clinical use [46].

The most popular type of material used to bond fixed retention was flowable composite; however, it can be supposed that the respondents did not consider it necessary to use a specialized composite dedicated to retention. Moreover, less than 10% used condensable composites, and 5.7% used chemically-cured composites for indirect bonding. The use of composite materials dedicated to bonding retainers confirmed knowledge of new specialized products; the wide use of flowable composite dedicated to dental restorations may be caused by high prices of orthodontic materials.

Retention with the use of fiber-einforced composite (FRC) was performed by approximately 30% of the respondents, mainly in periodontal patients. In addition, in these cases, a flowable composite was most often used to bond to the tooth surface. No questionnaire studies reporting on the use of different bonding materials for fixed retention could be found and used for discussion.

### 4.4. Limitations of the Present Study

A possible limitation of the present study may be associated with the fact that the *p* value indicating statistically significant differences between the groups analyzed does not prove any causality. However, analyzing causality was not the aim of the research. The authors intended to find the reasons behind which materials, and why, orthodontists use in everyday practice.

### 4.5. Summary

In summary, it can be stated that the group of Polish orthodontists was consistent in many aspects of orthodontic retention. The vast majority relied on double long-term retention with a long-term and regular follow-up appointments. They usually chose braided steel rectangular wire with a flowable composite as bonding material. However, doctors who used gold chain were most convinced by this type of retention wire, and they considered the other materials untrustworthy. A shortage of scientific-based knowledge regarding retention, follow-up period, and materials leads clinicians to choosing materials and protocols based on their clinical experience.

## 5. Conclusions

1. The protocols of orthodontic retention used by most Polish orthodontic practitioners relied on double (fixed and removable) long-term retention with regular follow-up appointments.
2. The most popular material is stainless steel braided rectangular wire bonded with a flowable composite.
3. Gold chain was the least popular. However, clinicians using gold chains are most convinced of only its clinical effectiveness, strongly rejecting other types of wire.
4. The use of round wire has garners the worst opinion among clinicians.
5. Fiber reinforced composite is less popular than other types of fixed orthodontic retention and is mostly used only in periodontal patients.
6. Stainless steel braided rectangular wire is the most comfortable for the dentist when it comes to adjustment and bonding, while the gold chain is the most stable after bonding.
7. Most clinicians believed that they could maintain perfect treatment results, but patients’ cooperation was a challenge.

## Figures and Tables

**Table 1:** The characteristics of the wires according to the respondents.

Answer Chosen	% of Answers for a Wire Type		
	MRW	BRW	GC
It is not always effective in preventing unwanted tooth displacement	36.5	15.4	8.7
Effectively prevents unwanted tooth displacement	8.7	44.2	26.9
It is easy to bend	27.9	48.1	17.3
It is hard to bend	10.6	11.5	1.9
It debonds often	14.4	5.8	1.9
It rarely debonds from the teeth	12.5	41.3	24
It is easy to bond	16.3	46.2	31.7
It is hard to bond	7.7	6.7	1.9
It deforms rarely	6.7	34.6	26
It deforms often	22.1	3.8	1.9
I have no opinion; I do not use it	41.3	24	60.6

MRW—multistranded round wire, BRW—braided rectangular wire, GC—gold chain.

**Table 2:** Bending characteristics of the wires according to the respondents.

Answer_vs_Answer		Reply	Respondents	Proportion	Reply	Respondents	Proportion	*p*
MRW is easy to bend	BRW is easy to bend	27	104	0.26	51	104	0.49	0.001 *
MRW is easy to bend	GC is easy to bend	27	104	0.26	18	104	0.173	0.178
BRW is easy to bend	GC is easy to bend	51	104	0.49	18	104	0.173	<0.001 *
MRW is hard to bend	BRW is hard to bend	11	104	0.106	11	104	0.106	>0.999
MRW is hard to bend	GC is hard to bend	11	104	0.106	2	104	0.019	0.022 *
BRW is hard to bend	GC is hard to bend	11	104	0.106	2	104	0.019	0.022 *

MRW—multistranded round wire, BRW—braided rectangular wire, GC—gold chain, *—statistically significant.

**Table 3:** Distribution of opinions on wire bonding.

Answer_vs_Answer		Reply	Respondents	Proportion	Reply	Respondents	Proportion	*p*
MRW is easy to bond	BRW is easy to bond	17	104	0.163	48	104	0.462	<0.001 *
MRW is easy to bond	GC is easy to bond	17	104	0.163	33	104	0.317	0.015 *
BRW is easy to bond	GC is easy to bond	48	104	0.462	33	104	0.317	0.047 *
MRW is hard to bond	BRW is hard to bond	6	104	0.058	6	104	0.058	>0.999
MRW is hard to bond	GC is hard to bond	6	104	0.058	2	104	0.019	0.279
BRW is hard to bond	GC is hard to bond	6	104	0.058	2	104	0.019	0.279

MRW—multistranded round wire, BRW—braided rectangular wire, GC—gold chain, *—statistically significant.

**Table 4:** Effectiveness of different wires in preventing unwanted tooth displacement according to the respondents.

Answer_vs_Answer		Reply	Respondents	Proportion	Reply	Respondents	Proportion	*p*
MRW is effective	BRW is effective	43	104	0.413	45	104	0.433	0.888
MRW is effective	GC is effective	43	104	0.413	28	104	0.269	0.041 *
BRW is effective	GC is effective	45	104	0.433	28	104	0.269	0.02 *
MRW is not effective	BRW is not effective	3	104	0.029	16	104	0.154	0.004 *
MRW is not effective	GC is not effective	3	104	0.029	9	104	0.087	0.137
BRW is not effective	GC is not effective	16	104	0.154	9	104	0.087	0.201

MRW—multistranded round wire, BRW—braided rectangular wire, GC—gold chain, *—statistically significant.

**Table 5:** Distribution of the opinions on which wire rarely debonds from the teeth.

Answer_vs_Answer		Reply	Respondents	Proportion	Reply	Respondents	Proportion	*p*
MRW detaches rarely	BRW detaches rarely	13	104	0.125	41	104	0.394	<0.001 *
MRW detaches rarely	GC detaches rarely	13	104	0.125	25	104	0.24	0.048 *
BRW detaches rarely	GC detaches rarely	41	104	0.394	25	104	0.24	0.025 *
MRW detaches often	BRW detaches often	13	104	0.125	6	104	0.058	0.149
MRW detaches often	GC detaches often	13	104	0.125	2	104	0.019	0.007 *
BRW detaches often	GC detaches often	6	104	0.058	2	104	0.019	0.279

MRW—multistranded round wire, BRW—braided rectangular wire, GC—gold chain, *—statistically significant.

**Table 6:** Susceptibility to deformation according to the respondents.

Answer_vs_Answer		Reply	Respondents	Proportion	Reply	Respondents	Proportion	*p*
MRW deforms rarely	BRW deforms rarely	7	104	0.067	35	104	0.337	<0.001 *
MRW deforms rarely	GC deforms rarely	7	104	0.067	27	104	0.26	<0.001 *
BRW deforms rarely	GC deforms rarely	35	104	0.337	27	104	0.26	0.289
MRW deforms frequently	BRW deforms frequently	21	104	0.202	4	104	0.038	0.001 *
MRW deforms frequently	GC deforms frequently	21	104	0.202	2	104	0.019	<0.001 *
BRW deforms frequently	GC deforms frequently	4	104	0.038	2	104	0.019	0.679

MRW—multistranded round wire, BRW—braided rectangular wire, GC—gold chain, *—statistically significant.

## Data Availability

All the raw data is available from corresponding author on a reasonable request.

## Supplementary Materials

The following supporting information can be downloaded at: https://www.mdpi.com/article/10.3390/ma15020666/s1, Supplementary S1: The original questionnaire in Polish. Supplementary S2: The raw spreadsheet of the answers of the respondents.
