# Supplementary material for: Orthodontic Retention—Protocols and Materials—A Questionnaire Pilot Study among Polish Practitioners
Source: Materials (Basel). 2022 Jan 16;15(2):666. doi: 10.3390/ma15020666 (PMC8779968; doi:10.3390/ma15020666)
Supplement: Supplementary file 1 [file materials-15-00666-s001.zip › Supplementary material S1.pdf]

1. Pański wiek *
Zaznacz tylko jedną odpowiedź.

a) <30
b) 30-40
c) 40-50
d) >50

2. Jaki rodzaj retencji stosuje Pan/i u pacjentów po zakończeniu leczenia ortodontycznego? *
Zaznacz tylko jedną odpowiedź.

a) Tylko stałą
b) Tylko wyjmowaną
c) Stałą oraz wyjmowaną

3. Według mnie okres retencji po zdjęciu aparatu stałego powinien trwać: *
Zaznacz wszystkie właściwe odpowiedzi.

a) Połowę okresu leczenia aktywnego
b) Tyle samo co leczenie aktywne
c) 2 razy dłużej niż aktywne leczenie
d) rok
e) 2 lata
f) 5 lat
g) Dożywotnio
h) Inne:

4. Częstość wizyt kontrolnych z aparatem retencyjnym w mojej praktyce to *
Zaznacz wszystkie właściwe odpowiedzi.

a) Co miesiąc
b) Co 3 miesiące
c) Co pół roku
d) Raz w roku
e) Pierwsza i druga wizyta co 3 miesiące, potem co pół roku
f) Pierwsza wizyta po miesiącu, druga po 3 miesiącach, potem co pół roku
g) Inne:

5.Jaką metodą klei Pan/i retainery? *
Zaznacz wszystkie właściwe odpowiedzi.

a) Pośrednio
b) Bezpośrednio

6. Jak ocenia Pan/i wyniki stosowanego leczenia retencyjnego? *
Zaznacz wszystkie właściwe odpowiedzi.

a) Utrzymanie idealnych pozycji zębów jest trudne.
b) Udaję mi się idealnie utrzymać wyniki fazy aktywnej leczenia u większości pacjentów
c) Awarie aparatów retencyjnych stanowią poważny problem kliniczny.
d) Awarie aparatów retencyjnych są maryginalnym problemem klinicznym.
e) Pacjenci zwykle współpracują podczas fazy retencyjnej leczenia ortodontycznego.
f) Pacjenci zwykle nie współpracują podczas fazy retencyjnej leczenia ortodontycznego.
g) Inne:

7.Jaki rodzaj retencji stałej Pan/i stosuje?
Zaznacz wszystkie właściwe odpowiedzi.

a) Włókno szklane (Fiber Reinforced Composite)
b) Drut stalowy

c) Drut tytanowy
d) Nie stosuję retencji stałej
e) Inne:

8. Jeżeli stosuje Pan/i włókno szklane, jakiego rodzaju jest to materiał?
Zaznacz wszystkie właściwe odpowiedzi.

a) Sznureczek
b) Taśma

9.Jeżeli stosuje Pan/i drut, jakiego rodzaju to materiał? *
Zaznacz wszystkie właściwe odpowiedzi.

a) Stalowy pojedynczy
b) Stalowy pleciony okrągły
c) Stalowy pleciony czworokątny
d) Tytanowy
e) Złoty łańcuszek
f) Niklowo-tytanowy
g) Inne:

10.Jeżeli stosuje Pan/i drut, jakie ma on wymiary?
Zaznacz wszystkie właściwe odpowiedzi.

a) 0.014 × 0.014
b) 0.015"
c) 0.016
d) 0.016″ × 0.022″
e) 0.0175'
f) 0.0195
g) 0.027"
h) Nie wiem
i) Inne:

11.Jaki materiału używa Pan/i do klejenia drutów retencyjnych? *
Zaznacz wszystkie właściwe odpowiedzi.

a) Płynnego materiału kompozytowego dedykowanego do retencji
b) Płynnego materiału kompozytowego do wypełnień
c) Materiału kompozytowego przeznaczonego do wypełnień
d) Kleju światłoutwardzalnego do zamków ortodontycznych
e) Materiału światłoutwardzalnego przeznaczonego do klejenia pośredniego
f) Materiału chemoutwardzalengo przeznaczonego do klejenia pośredniego
g) Inne:

12.Pana/i zdaniem drut retencyjny okrągły *
Zaznacz wszystkie właściwe odpowiedzi.

a) Jest łatwy do doginania
b) Rzadko się odkleja
c) Łatwo się zakłada
d) Rzadko się deformuje
e) Skutecznie zapobiega niepożądanym przesunięciom zębów
f) Jest trudny do doginania
g) Często się odkleja
h) Trudno się zakłada
i) Często się deformuje
j) Nie zawsze skutecznie zapobiega niepożądanym przesunięciom zębów
k) Nie mam zdania - nie stosuję

13.Pana/i zdaniem drut retencyjny czworokątny *

Zaznacz wszystkie właściwe odpowiedzi.

a) Jest łatwy do doginania
b) Rzadko się odkleja
c) Łatwo się zakłada
d) Rzadko się deformuje
e) Skutecznie zapobiega niepożądanym przesunięciom zębów
f) Jest trudny do doginania
g) Często się odkleja
h) Trudno się zakłada
i) Często się deformuje
j) Nie zawsze skutecznie zapobiega niepożądanym przesunięciom zębów
k) Nie mam zdania - nie stosuję

14. Pana/i zdaniem retencyjny złoty łańcuszek *
Zaznacz wszystkie właściwe odpowiedzi.

a) Jest łatwy do doginania
b) Rzadko się odkleja
c) Łatwo się zakłada
d) Rzadko się deformuje
e) Skutecznie zapobiega niepożądanym przesunięciom zębów
f) Jest trudny do doginania
g) Często się odkleja
h) Trudno się zakłada
i) Często się deformuje
j) Nie zawsze skutecznie zapobiega niepożądanym przesunięciom zębów
k) Nie mam zdania - nie stosuję

15.Włókno szklane do retencji *
Zaznacz wszystkie właściwe odpowiedzi.

a) Nie stosuję
b) Stosuję u pacjentów z chorobą przyzębia po leczeniu ortodontycznym
c) Stosuję u większości pacjentów po leczeniu ortodontycznym
d) Stosuję u wszystkich pacjentów
e) Inne:

16. Pana/i zdaniem włókno szklane:
Zaznacz wszystkie właściwe odpowiedzi.

a) Jest estetyczne
b) Jest trwałe
c) Łatwo się zakłada
d) Rzadko się deformuje
e) Skutecznie zapobiega niepożądanym przesunięciom zębów
f) Utrudnia higienę
g) Często się odkleja
h) Trudno się zakłada
i) Często się deformuje
j) Nie zawsze skutecznie zapobiega niepożądanym przesunięciom zębów
k) Nie mam zdania - nie stosuję

17. Jaki materiału używa Pan/i do klejenia szyn z włókna szklanego? *
Zaznacz wszystkie właściwe odpowiedzi.

a) Płynnego materiału kompozytowego dedykowanego do retencji
b) Płynnego materiału kompozytowego do wypełnień
c) Materiału kompozytowego przeznaczonego do wypełnień

d) Kleju światłoutwardzalnego do zamków ortodontycznych
e) Materiału światłoutwardzalnego przeznaczonego do klejenia pośredniego
f) Materiału chemoutwardzalengo przeznaczonego do klejenia pośredniego
g) Inne:
